# Knowledge Graph Applications in Medical Imaging Analysis: A Scoping Review

**DOI:** 10.34133/2022/9841548

**Published:** 2022-06-14

**Authors:** Song Wang, Mingquan Lin, Tirthankar Ghosal, Ying Ding, Yifan Peng

**Affiliations:** 1 The University of Texas at Austin, Austin, USA; 2 Population Health Sciences, Weill Cornell Medicine, New York, USA; 3 Institute of Formal and Applied Linguistics, Charles University, Czechia, Czech Republic

## Abstract

**Background.:**

There is an increasing trend to represent domain knowledge in structured graphs, which provide efficient knowledge representations for many downstream tasks. Knowledge graphs are widely used to model prior knowledge in the form of nodes and edges to represent semantically connected knowledge entities, which several works have adopted into different medical imaging applications.

**Methods.:**

We systematically searched over five databases to find relevant articles that applied knowledge graphs to medical imaging analysis. After screening, evaluating, and reviewing the selected articles, we performed a systematic analysis.

**Results.:**

We looked at four applications in medical imaging analysis, including disease classification, disease localization and segmentation, report generation, and image retrieval. We also identified limitations of current work, such as the limited amount of available annotated data and weak generalizability to other tasks. We further identified the potential future directions according to the identified limitations, including employing semisupervised frameworks to alleviate the need for annotated data and exploring task-agnostic models to provide better generalizability.

**Conclusions.:**

We hope that our article will provide the readers with aggregated documentation of the state-of-the-art knowledge graph applications for medical imaging to encourage future research.

## 1. Introduction

In recent years, incorporating structured domain knowledge into downstream tasks has drawn great research attention from industry and academia [1]. This is because domain knowledge provides a proper understanding of a field which can be represented as a knowledge graph that can facilitate efficient inference to empower downstream tasks.

A knowledge graph represents the actual facts in the form of structured graphs, including entities (e.g., realistic objects and general concepts) and the relationships between entities [1]. It provides semantically structured information that computers can interpret and promises to build more intelligent systems to solve numerous real-world problems.

**Figure 1:**
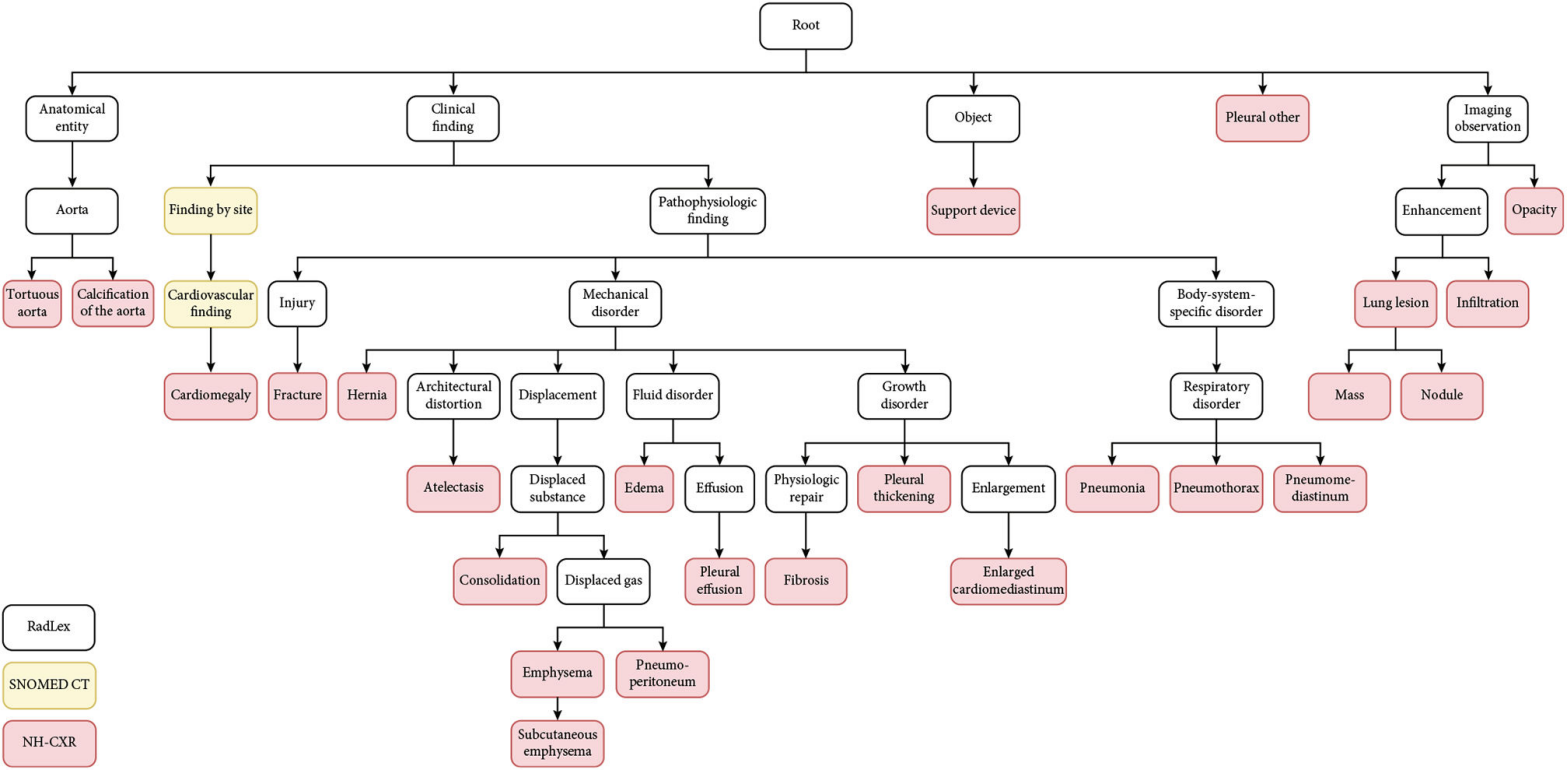
Radiology knowledge graph example: NIH Chest X-ray labels based on RadLex and SNOMED_CT.

Knowledge graphs (viewed as the graph structure) differ from knowledge bases in terms of the involvement of formal semantics for interpretation and inference over facts (Figure 1). Knowledge graphs (KGs) like DBPedia [2], NELL [3], and Wikidata knowledge base [4] have become instrumental in various machine learning applications, such as information retrieval [5], information extraction [6-8], question answering [9, 10], and recommendation [11-13].

Within a biomedical setting, researchers can utilize knowledge graphs to tackle various realistic problems, for example, aiding efforts to diagnose patients [14], exploring possible disease treatments [15, 16], and identifying associations between biomolecules and diseases [17]. Oftentimes, solutions require a process called representation learning, which is to learn the mappings between the knowledge graphs and low-dimensional graph representations in feature space [1]. The representation learning process aims to encode the local as well as the global structure of a knowledge graph and map it to an embedding that can be utilized by algorithms for downstream tasks. Among various knowledge graph applications in biomedicine, medical imaging (e.g., radiography, ultrasound, and magnetic resonance imaging) serves as one of the most significant diagnostic aids that are readily available to physicians [18].

Deep learning methods try to train algorithms to identify abnormal regions and tissue variations in a manner similar to human beings [19]. Medical record histories, previous diagnoses made by pathologists or radiologists, are used to train the algorithms. The algorithm learns this with large amounts of data, and after analyzing thousands of iterations of different images and diagnoses, it eventually will learn to make some diagnoses. In the medical imaging analysis domain, knowledge graphs have drawn a lot of research attention. Though there are comprehensive survey papers for knowledge graph applications in biomedical informatics [20], we have not seen any literature survey for knowledge graph applications in medical imaging analysis. To bridge this gap, we conducted a systematic review on knowledge graph applications in medical imaging analysis. According to our review, most studies applied knowledge graphs to specific topics such as disease detection, localization, and report generation. In this work, we describe various knowledge graphs applications in medical imaging analysis and then point out future directions that have yet to be explored.

## 2. Methods

First, we will introduce the background of knowledge graphs. Second, we will describe the data sources and search strategies, including the inclusion and exclusion criteria, and how we selected the articles. At last, we will talk about how we conducted data synthesis and analysis.

### 2.1. Knowledge Graphs in General.

Recently, knowledge graphs have become a predominant part of many information systems where structured prior knowledge is needed. The concept of graphical knowledge representation can be traced back to 1956 when Richens proposed the idea of Semantic Net [21]; however, the community realized the importance of his work only belatedly. MYCIN [22] features a knowledge base containing about 600 rules and is one of the most well-known rule-based expert systems for medical diagnosis. Many researchers promoted the idea of graph-based knowledge representation aiming to assemble human knowledge. Furthermore, Resource Description Framework (E. [23]) and Web Ontology Language [24] were released and became the mainstay of Semantic Web.

In 2009, the concept of Linked Data was proposed to build the links between different datasets in the Semantic Web with each other and treat it as one global knowledge graph [25]. Subsequently, various ontologies or knowledge bases were published, such as WordNet ([26]), YAGO [27], DBpedia [2], and Freebase [28], to realize the idea of structured knowledge representation in the form of a graph. In 2012, Google proposed Knowledge Graph (Knowledge Vault) to utilize semantic knowledge in the application of web search, and the concept gained great popularity [29]. Google uses the knowledge graph to help identify and disambiguate entities in texts, utilize semantically structured summaries to enrich the search results, and further provide links to related entities in exploratory search [29]. Recently, many companies such as Microsoft, Amazon, and Pinterest have started investing massive resources to build knowledge graphs for their commercial applications [30-32].

### 2.2. Knowledge Graphs for Medical Imaging Analysis.

Machine learning techniques have recently been applied to all stages of radiotherapy, from diagnostic imaging, using image registration for risk delineation, to the automated planning and outcome assessment [33, 34]. To provide quality healthcare, such as custom medicine or treatment planning refinement, machine learning techniques are able to offer assistive insights. Bringing together artificial-intelligence-driven (AI-driven) radiotherapy and deep-learning-based medical imaging analysis is a promising direction. To provide precision radiotherapy, analyzing medical images and other modalities to derive representative features in a quantitative manner is vital. There tends to be significantly more information underlying in images and other data modalities that cannot be visually perceived in a straightforward way. However, sophisticated algorithms enable us to mine and use these underlying information to improve diagnosis and treatments. There are two main reasons why AI-based radiotherapy is expected to outperform conventional radiotherapy. First, many latent features that cannot be perceived by human readers can be utilized by analyzing radiomics in an automated manner. Moreover, we can mine and infuse priori in a data-driven and end-to-end manner, making AI-based radiotherapy more powerful [34]. The radiotherapeutic process can generate a large amount of data on anatomical, metabolic, etc. [35]. One major challenge is that it can be complicated to extract and present those data in a meaningful yet interpretable manner. Moreover, medical reports are oftentimes written in natural languages, where the report sensitivity and specificity, associated decisions need to be handled appropriately to provide better patient treatments than the current standards. One ideal solution is to synergize knowledge graphs to represent a generalization of prior knowledge from different modalities for treatment planning optimization or patient prognosis improvements [34].

Building a knowledge graph utilizing a patient’s electronic medical records and reports can provide valuable information for disease reasoning and further treatment planning. NLP techniques can be very helpful for identifying and extracting knowledge from text inputs. When given the medical reports, we can use online services such as the Watson Natural Language Understanding platform or the Amazon Comprehend Medical to construct the graph rather than from scratch. Based on these cloud services and other systems, we can distill and query high-quality domain-specific rules, knowledge graphs from unstructured or semi-structured contents extracted from images and data such as medical conditions, medication details (dosage, strength, and frequency), and other data like doctors’ notes, clinical reports, and patient health records [34].

With the efforts mentioned above, we will have treatment-related feature graphs and knowledge graphs from medical images and medical text data. These two types of graphs are in different domains: one from images and data biologically/clinically informative, and the other is in terms of professional languages directly interpreted. Therefore, we need to bridge these two domains via an across-domain graph transformation. To this end, we can use a graph-based encoder-decoder network, including graph convolution, graph pooling. The encoder will extract the information from a radiomic graph, while the decoder will reconstruct a corresponding graph. The bottleneck between the encoder and the decoder will bridge the image and text domains. In the cases of dynamic changes with different reports, one can use a graph-based RNN to learn a dynamic graph mapping.

### 2.3. Data Sources and Search Strategies.

Following the Preferred Reporting Items for Systematic Reviews and Meta-Analyses (PRISMA) guidelines, we conducted a comprehensive search of English-language articles published between 2006 and 2021 from five databases. The databases include IEEE Xplore (https://ieeexplore.ieee.org/Xplore/home.jsp), PubMed (https://pubmed.ncbi.nlm.nih.gov/), Arxiv (https://arxiv.org/), Google Scholar (https://scholar.google.com/), and the ACM Digital Library (https://dl.acm.org/). The search strategy is to iteratively search keywords for relevant articles and related citations. As shown in Table 1, the keywords used to retrieve literature included knowledge graph(s) and medical imaging, knowledge graph(s) and medical image(s), graph(s) and medical imaging, graph(s), and medical image(s).

### 2.4. Article Selection.

After the acquisition of potential articles, we conducted abstract and full text screening. Article exclusion criteria included the following: duplicates; article types such as conference abstract, review, editorial, erratum, letter, note, and comment; unavailable full text; and articles irrelevant to knowledge graph applications in medical imaging analysis. The inclusion criteria for the target publications were as follows: (1) knowledge graphs were used and (2) the aim was to solve medical imaging analysis problems. Two reviewers used the eligibility criteria to screen the articles. During the screening process, all conflicting opinions among reviewers were discussed until we reached a consensus.

### 2.5. Data Synthesis and Analysis.

From the selected articles, our data synthesis was motivated by an approach to gain insight into how knowledge graphs were applied to different medical imaging analysis tasks and review how knowledge graphs contributed to the medical imaging analysis tasks. We began by examining the general characteristics of the included studies, such as the publication year trend, publication country, and the focus medical imaging analysis topic. Furthermore, we investigated the datasets utilized by the included studies, to provide readers with an insight into the available data sources that can be used for different medical imaging analysis topics.

## 3. Results

### 3.1. Identification of Included Studies.

**Figure 2:**
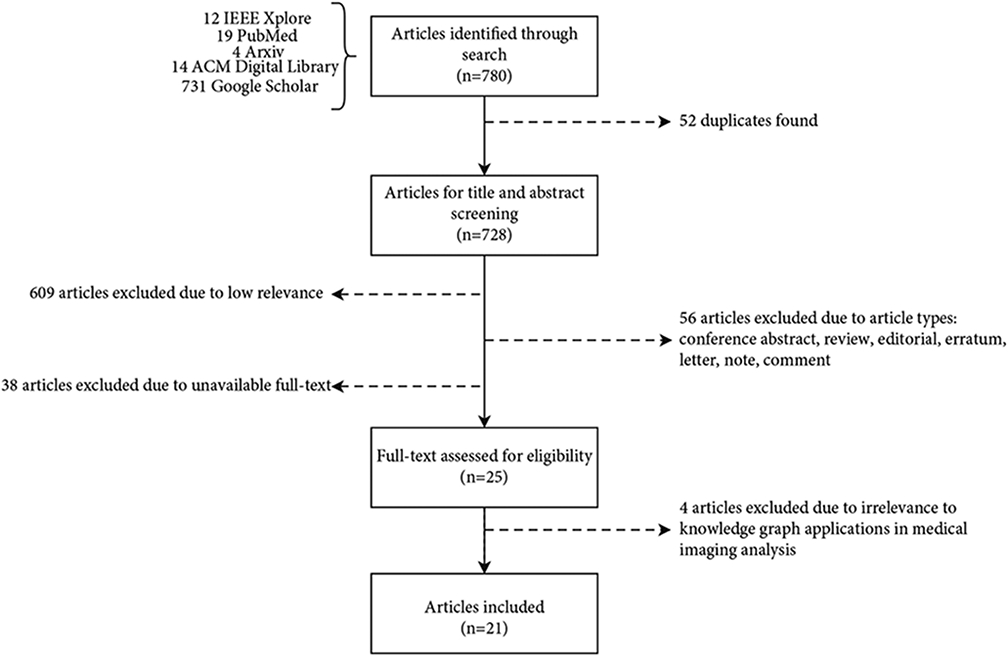
The flowchart of the article selection process.

We retrieved 780 articles from five databases, of which 728 articles were found to be unique. The titles and abstracts of articles were screened for article filtering and selection. Articles were sorted based on the relevance to applying knowledge graphs to medical imaging analysis, where 609 articles were excluded due to low relevance. We excluded several article types, including the conference abstract, review, editorial, erratum, letter, note, and comment, resulting in excluding 56 articles. We further excluded 38 articles without full text. 25 articles remained for subsequent full-text reviews. During the full-text screening, 4 articles were excluded due to irrelevance to knowledge graph applications in medical imaging analysis. After this full-text screening process, 21 articles were selected to be included in this scoping review. The article selection flowchart is shown in Figure 2.

### 3.2. Statistical Characteristics of the Included Articles.

**Figure 3:**
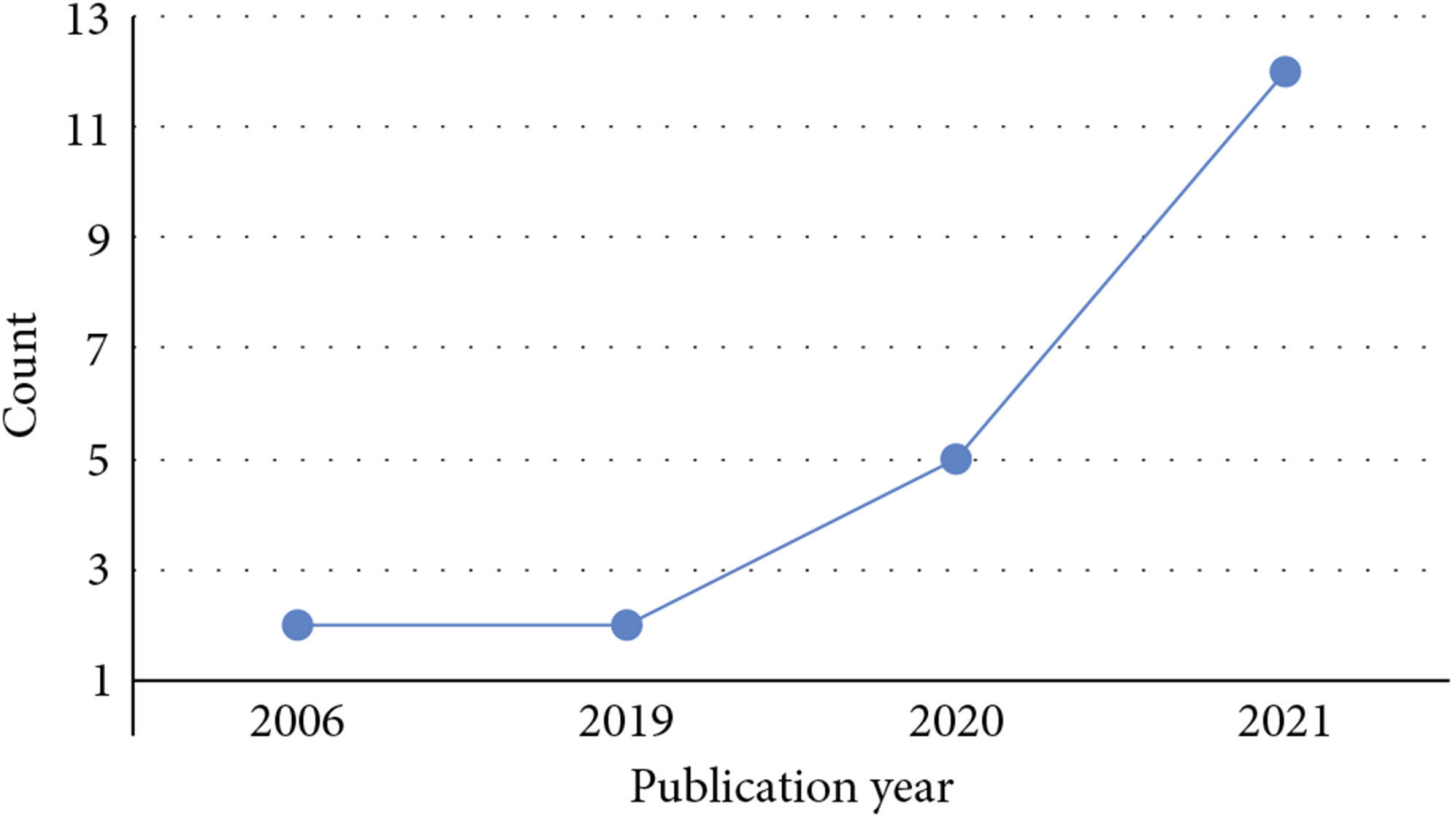
Year trend of reviewed articles.

**Figure 4:**
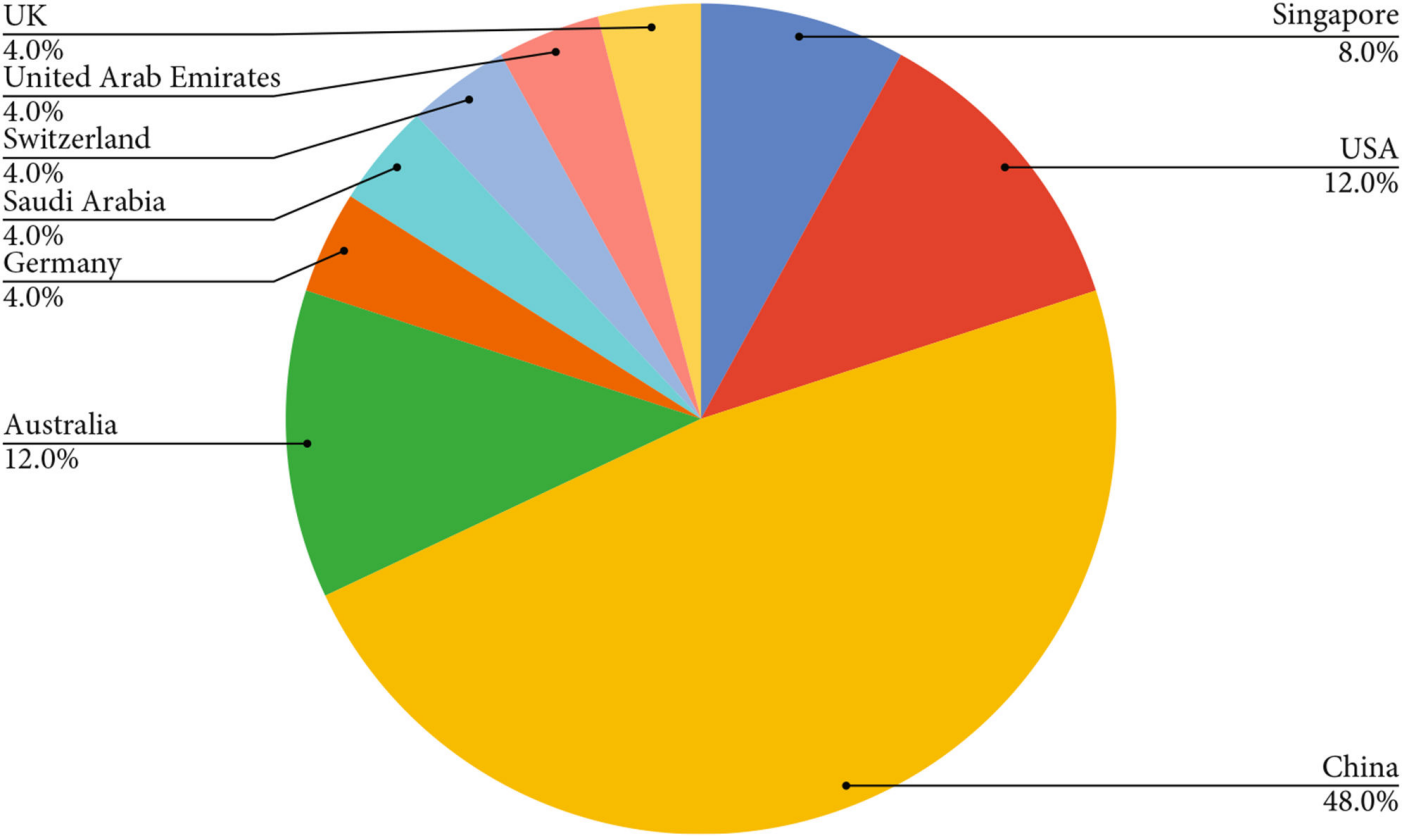
Publication country distributions.

**Figure 5:**
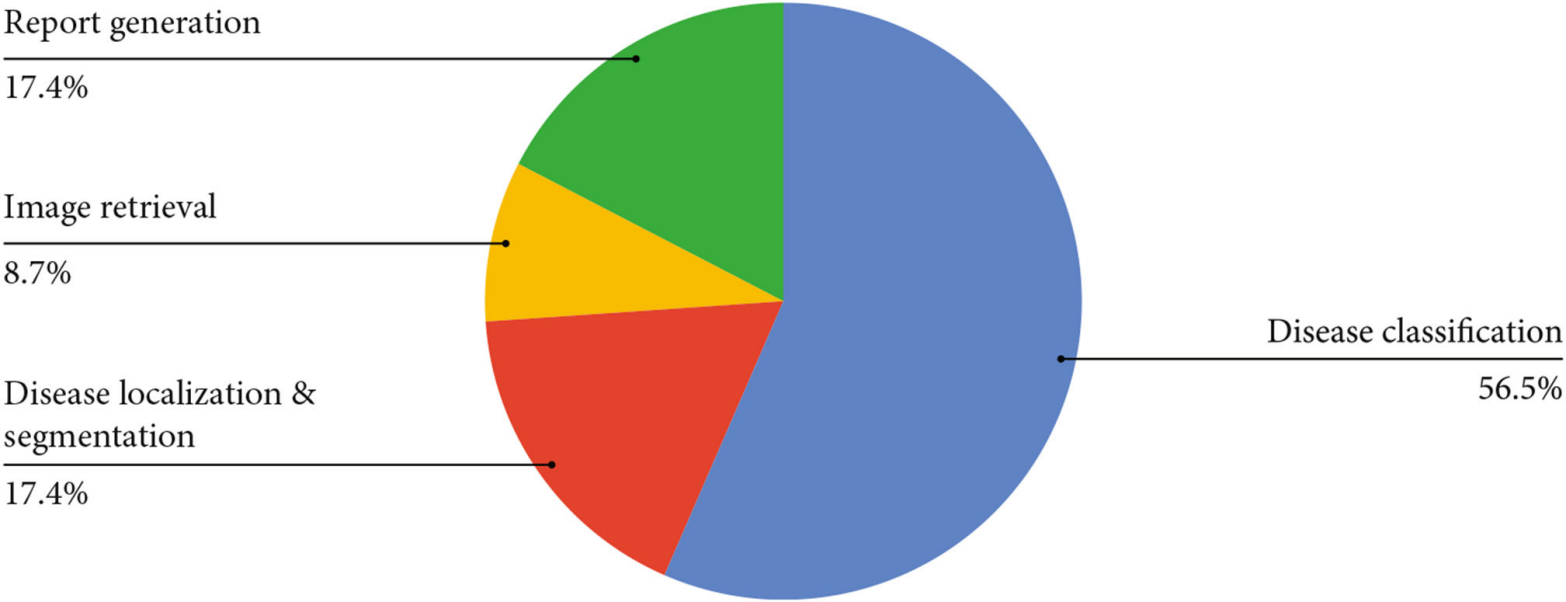
Application topic distributions.

All the 21 articles included in this work are published from 2006 to 2021, with a noticeable increment in the number of papers published per year (Figure 3). The included publications are across nine countries, with most contributions coming from China (48%) (Figure 4). Among all the included articles, the most common application of knowledge graphs in medical imaging is disease classification (56.5%), followed by disease localization and segmentation (17.4%), report generation (17.4%), and image retrieval (8.7%) (Figure 5).

### 3.3. Disease Classification.

One of the most common computer vision tasks is to classify images into appropriate categories [36]. Disease classification is especially of vital importance in medical imaging to assist diagnosis [18]. The types and sizes of image datasets are increasing dramatically. Hence often, we need to classify images from unseen classes into the correct categories based on the relationships between the seen and unseen classes. Our world contains millions of visual concepts. Due to its complex and dynamic characteristics, it is impossible to build a large dataset for every concept to ameliorate various computer vision tasks. Prior knowledge is the key to building semantic relationships between classes, which can be of great help, especially when we have limited training data. Knowledge graphs contain rich knowledge, modeling the relationships among classes or concepts. Incorporating disease classification in medical imaging with knowledge graphs has been explored by researchers and has shown promising results [18]. Table 2 lists the overview of datasets used by the included articles related to disease classification in this work. The most commonly used datasets are IU X-Ray [37], NIH Chest X-Ray/ChestX-Ray 14 [38], and CheXpert [39], and they all include medical images, associated medical reports, and disease labels.

There are five articles included in this review that explored binary disease classification incorporated with knowledge graphs. Xie et al. constructed a knowledge-based collaborative sub-model for the task of nodule classification. They proposed to fine-tune three pretrained ResNet-50 networks using three types of image patches. The three pretrained ResNet-50 networks were, respectively, used to characterize the nodules’ overall appearance, voxel, and shape heterogeneity [40]. In this way, a knowledge-based collaborative model was introduced to incorporate the multiview information for separating the benign nodules from the malignant ones using very limited data. Yu et al. aimed to facilitate the process of pneumonia diagnosis [41]. A graph-based feature reconstruction module was employed that takes the produced image features from a trained convolutional neural network (CNN) as input. The resulting combined features will be fed to a one-layer graph neural network (GNN) to classify chest X-ray images into two classes: normal and pneumonia. According to Chen et al., most existing work manually built a population graph for structural information aggregation where the relationship between nodes was represented by the graph adjacency matrix [42]. Chen et al. automatically constructed the population graph and further utilized the fusion of multimodal information, which improved the diagnostic accuracy for Autism Spectrum Disorder and breast cancer. Specifically, they proposed an encoder that can select the appropriate phenotypic measures in an automatic manner in terms of their spatial distribution. They further computed the edge weights between nodes utilizing a mechanism which is aware of the text similarity. Liu et al. claimed to outperform previous work on the Mammogram mass classification task [43]. To model the intrinsic geometric and semantic relations of ipsilateral views, they proposed a Bipartite Graph Convolutional Network. The asymmetric visual information of bilateral views was widely adopted in clinical practice to assist the diagnosis process of lesions. To model the structural similarities of bilateral views, an Inception Graph Convolutional Network was further proposed. The representations learned from the constructed graphs were capable of multiview reasoning, since there was a systematical propagation of the multiview information through nodes [43]. Fu et al. pointed out that most existing methods only focus on the image modality while ignoring or not fully leveraging information from other modalities [44]. They proposed to exploit the inter-category relationships in the 7-point visual category checklist (7PC) for Melanoma diagnosis. Specifically, they proposed to use a graph-based relational module to leverage inter-categorical and inter-modal relations. The dermoscopy visual structure details were further prioritized by representing the features in a graph network [44]. Another category embedding learning module was also employed to capture the specialized representations for each category and support the graph-based relational module.

Six articles explored multilabel classifications in medical imaging. Zhang et al. constructed a disease finding knowledge graph and utilized it to tackle the disease classification task [45]. Incorporating a knowledge graph with the disease classification task allowed for dedicated feature learning for each disease finding [45]. Similarly, Hou et al. modeled the correlations among disease labels by employing the graph convolutional network (GCN). They further pretrained the disease label embeddings on the radiology reports. A transformer-based encoder was employed to fuse the semantic features along with the encoded image features to initialize the graph features [46]. To have a better graph representation capability, they mined additional medical terms from radiology reports, and these newly mined terms were added to the graph serving as auxiliary nodes without changing the actual output space size.

However, Zhou et al. pointed out that the developing a robust automated diagnosis system could be hindered by the fact that the lesions can have inconsistent appearances and high complexities in chest X-rays [47]. They proposed one promising approach to address this issue, which is to attend to the abnormal regions and exploit relevant prior information [47]. To have a better thoracic disease identification performance, especially for those whose lesions rarely appear on both sides symmetrically, one contrastive network was proposed to learn the intra-attentive abnormal features between the left and right lung. They further utilized an inter-contrastive attention model to acquire the abnormal attention map. Specifically, they compared the query scan with multiple anchor scans where no lesions were present. After the features were weighted using the intra- and inter-contrastive attention scores, the radiology graph was further constructed for graph reasoning in a dual-weighted manner in addition to the basic visual-spatial convolution [47]. Following the same direction, Agu et al. noted that most existing methods solely used the chest X-ray images for classification, but they failed to utilize the underlying anatomical information that can be really helpful [48]. They utilized a GCN which enables their model to learn the anatomical region relationships and label dependencies in the chest X-ray images. They further created an anatomical region adjacency matrix based on the correlation of the labels across different regions. Combining this with a detection module, they proposed a multilabel chest X-ray classifier that can classify image findings and localize them to their anatomical regions [48].

According to Sekuboyina et al. [49] learning to map images to binary labels made it a challenging task to take advantage of auxiliary information (e.g., annotation uncertainty, and label dependencies). A multimodal knowledge graph was constructed using chest X-ray images and their labels. They approached the task of multilabel disease classification in a link prediction manner. They claimed that they added additional nodes and relations to incorporate auxiliary information into the graph [48]. Similarly, Chen et al. noted that given the fact that graph data featured high complexity, most previous works failed to fully use such valuable graph-structured information, but solely focused on learning to classify the input into binary labels [50]. As a result, they proposed to explicitly explore the graph structure information, such as the pathology dependencies, for the classification task. They introduced the pathology word embeddings and multilayer graph information propagation to generalize the relationships between pathologies into a set of classifier scores. The flexible integration into the image feature embedding module and the adaptive recalibration of multilabel outputs with these scores were made possible during the training process [50].

Since 2020, knowledge graphs have also been explored in COVID-19–related research and shown noticeable performance improvements. Zheng et al. pointed out that current deep learning methods suffered from data adequacy issues and that multimodal information should be considered together to make accurate inferences [51]. To solve this, they proposed a multimodal graph attention embedding mechanism to assist diagnosing COVID-19. Their method learned the relational embeddings in a constituted knowledge graph and, at the same time, improved the classifier through the medical knowledge attention mechanism [51]. According to Mudiyanselage et al., the poor performance for unseen data in COVID-19 classification can result from the limited correlation between the pretrained model and a specific imaging domain (e.g., X-ray) and the possibility of overfitting [52]. They proposed that the relational knowledge between data instances can be exploited through graph representations and further utilized through graph convolutions [52].

To summarize, the aforementioned work utilized different types of knowledge graphs, and they incorporated the knowledge graphs with the disease classification task using three approaches: (1) embed visual features to preconstructed prior knowledge abnormality graph [44-50], region graph [40, 43], pathology graph [50], and population graph [42] and (2) extract and use visual features as graph nodes [41, 52]; (3) use images and/or text descriptions of diagnose as graph nodes [49, 51]. Though these work applied knowledge graphs in various ways, the results showed that incorporating knowledge graphs with disease classification achieved noticeable classification performance boosts; for example, Zhang et al. achieved 1.4% improvement on average AUC, 4.7% AUC improvement on cardiomegaly, and 4.5% AUC improvement on atelectasis after adding knowledge graphs to the baseline DenseNet [39] model [45]. Zhou et al. achieved a 3.77% improvement on average AUC when incorporating disease identification with prior knowledge on the NIH Chest X-ray dataset and a 3% average AUC improvement on the CheXpert dataset [47].

### 3.4. Disease Localization and Segmentation.

In medical imaging, disease localization and segmentation are useful for clinical diagnosis, disease assessment, and treatment planning [64]. Previous supervised methods suffered from the lack of finely annotated data, and weakly supervised methods often generated inaccurate or incomplete regions [65]. When taking into account the anatomical region relationships and the correlations between images, complementary information can be obtained to improve the disease localization accuracy. This also aligns with the clinical practice in the medical domain: usually to train a radiologist to analyze X-ray images, they read many X-ray images and compare the differences between different images and even the differences between different regions of the same image [65]. Infusing knowledge graphs into the system offers the potential for more accurate localizations and segmentations. Table 3 lists the overview of included articles and the datasets that are used. The most commonly used datasets for this task are CheXpert [39] and NIH Chest X-Ray/ChestX-Ray 14 [38]. Both datasets include medical images and associated medical reports and disease labels.

Peng et al. identified the fissure region of interest using lung anatomy prior knowledge and then isolated the plate-like structures from clutters utilizing an oriented derivative of stick filter for lobar fissure verification. Finally, to segment lung lobes, they completed the incomplete fissure surface employing a surface fitting model [66]. Qi et al. noted that one reason for incomplete localization regions was neglecting the anatomical region relationships within each image and the inter-image relationships [65]. Hence, they proposed to model the inter-image relationships by comparing multiple images in an inter-image graph and to model the intra-image relationships by comparing different regions in an intra-image graph. These cross-image and cross-region relationships were used as the contextual and compensating knowledge and were incorporated for disease localizations. Through ablation study, they showed that the model employing the intra-image and inter-image prior knowledge outperformed the localization accuracy of the baseline model by 0.08, 0.11, and 0.1 when the intersection over union (IoU) threshold was 0.3, 0.5, and 0.7. Zhao et al. also noted that most weakly supervised disease localization methods failed to consider the chest X-ray image characteristics (e.g., the highly structural attributes) [67]. They used a very similar method to Qi et al. [65], which integrated the intra-image anatomical structural knowledge and inter-image knowledge into one unified framework.

In summary, the aforementioned articles incorporated knowledge graphs with disease localization and segmentation using two approaches: (1) embed visual features to preconstructed prior knowledge region graph [65-67]; (2) use images as graph nodes [65, 67]. These articles identified the importance of prior knowledge, proposed to infuse prior knowledge into the disease localization and segmentation tasks in the form of knowledge graphs, and showed that the prior knowledge did bring drastic performance improvements.

### 3.5. Report Generation.

Natural language captioning aims to summarize visual information in one sentence or generate one topic-related paragraph [70]. Medical report generation translates the medical images to human-readable medical reports, which requires an increased capability to cover accurate abnormal terminologies, understand the medical domain knowledge, and describe the findings at a semantic-coherent and fine-grained level that should satisfy both medical commonsense and logic [71]. Outstanding challenges associated with automatic medical report generation lie in successfully detecting visual groundings and incorporating medical domain knowledge. To write a medical image report, radiologists will first check a patient’s images, carefully inspect the abnormal regions to identify the findings, and then describe the abnormal findings in detail based on prior medical experiences and medical knowledge. Only employing the global images as input and training the language model with the dataset’s corpora alone cannot provide the underlying prior knowledge vital for accurate reporting. Several works infused knowledge graphs into report generation and showed the performance gain on the quality of generated reports. The datasets used by the included articles related to report generation are listed in Table 4, and the most used dataset is IU X-Ray [37].

Zhang et al. constructed a graph embedding module on multiple disease findings as the prior knowledge to assist report generation. The incorporation of a knowledge graph allowed for dedicated feature learning for each disease finding and the relationship modeling [45]. The knowledge graph module improved the baseline SentSAT model [72] on nearly all report generation evaluation metrics, especially 0.036 improvements on the CIDEr metric. Li et al. [73] noted the significant challenges towards bridging visual and linguistic modalities; hence, they proposed to encode visual features as an abnormality knowledge graph, which incorporated the visual features with prior medical knowledge, and was then used to guide the report template retrieval-paraphrase process or used for disease classification. Similarly, Liu et al. [43] noted that visual and textual data biases remained a challenge for data-driven report generation systems, so in addition to the disease-tag attended visual features and the disease-attended textual features, they proposed to explore the prior knowledge from a predefined medical knowledge graph guided by attended-image features, and further adaptively distill the knowledge for report generation. Through ablation study, they show that removing the prior knowledge graph module from the proposed model will cause a significant drop in all evaluation metrics, especially 0.66 drop on CIDEr, 0.34 drop on BLEU-1. Li et al. pointed out that previous methods suffered from the deviation that taught models to generate inessential sentences regularly. Therefore, inspired by Generative Pre-Training, they proposed to guide medical knowledge transfer and learning through a medical graph encoder, by integrating internal visual feature fusion and external medical linguistic information [71].

To summarize, the aforementioned articles incorporated knowledge graphs with report generation using one common approach: embed visual features to preconstructed prior knowledge abnormality graph [43, 45, 71, 73, 74]. Knowledge graphs utilized in the included articles modeled the relationships of disease findings and bridged multiple modalities by embedding visual representations. Through ablation studies, all included articles demonstrated the effectiveness of incorporating knowledge graphs with report generation.

### 3.6. Image Retrieval.

Automated image retrieval systems show enormous potential in medical applications [76]. It can be beneficial for the clinical decision-making process to extract similar images that share common aspects (i.e., modality, anatomic region, and disease). This allows for extracting similar images with similar diagnoses and also allows for finding similar images but with different diagnoses. In medical domain, doctors can adopt image retrieval systems to retrieve images with known pathologies that are similar to the anchor image and further to assist the diagnosis process. In addition to doctors, medical researchers, lecturers, and even students can extract relevant images using visual retrieval methods for their teaching and research. Primitive features (i.e., color and texture) are still the dominant features used by most image retrieval systems for image presentation purpose [77]. No medical knowledge was used in this process; hence, there exists the domain gap if we want to apply the systems to medical domain. This loss of information can be reduced by incorporating prior knowledge and other sources of knowledge [78]. Table 5 lists the overview of datasets used in the image retrieval articles included in this review.

Lacoste et al. presented their medical image retrieval method incorporating medical prior through a fusion framework [79]. The text knowledge infused was from the Unified Medical Language System (UMLS) sources. They learned semantic features from examples and further to derive the visual knowledge. UMLS concepts enabled the communication between visual and textual information, allowing a higher level systematic medical data standardization. Racoceanu et al. employed similar methods by using global indexing to access image modality and local indexing to access local semantic features to fuse the textual and visual knowledge into image retrieval [80].

In summary, these two included articles incorporated knowledge graphs with image retrieval using one common approach: represent images and texts in UMLS graphs [79, 80]. The introduced knowledge graphs facilitated the communication between multiple modalities and benefitted the image retrieval task.

## 4. Discussion

After the article selection phase, there were 21 articles selected and included in this survey. This relatively small number of articles related to knowledge graph applications in medical imaging analysis may suggest the cross-disciplinary gaps and lack of collaborations. In this section, we identified the limitations of the included articles and suggested the potential future directions.

### 4.1. Disease Classification.

Most articles share some common limitations in this review. The datasets are still too small in size (on average, 129,788 images, ranging from 450 to 384,580) to provide results that are more convincing. The construction of graphs and the reconstruction of features are important aspects of most of the works. However, the graphs were constructed on a given dataset, making the extension to other domains inconvenient and challenging. For example, some graphs were designed as components of the proposed model for diagnosing chest diseases, which would not work for a brain tumor diagnosis task. If researchers want to transfer the method to handle a problem in another domain, building a new graph using a similar approach would be necessary. Also, an encoding component pretrained towards a specific task (multilabel classification) could result in representations that do not generalize well across tasks. Furthermore, global classifications can be unreliable even when the predicted label is correct. The classifier might predict the correct label but for a wrong reason at an irrelevant spot.

For the future direction, one can consider a semisupervised learning framework to reduce the demand for annotated data. Also, we can think of considering more sophisticated graph structures, which model more detailed disease relationships in the future. Other approaches can be explored to better incorporate visual and semantic features. It is worth exploring a task-agnostic representation learning framework for better generalizability. Another future research direction is to combine the encoding and embedding modules resulting in a fully end-to-end formulation.

### 4.2. Disease Localization and Segmentation.

The included articles regarding disease localization and segmentation in this review did not consider the label uncertainty, which is worth exploring to improve the performance. The reported results showed that small targets (e.g., atelectasis, effusion, and nodule) were more challenging to localize due to their relatively smaller size. Algorithms applicable to the localization of small targets (e.g., atelectasis, effusion, nodule) are worth exploring.

Existing work like Peng et al. heavily depends on other tasks like airway segmentation. However, the segmentation is an arduous task as it is highly sensitive to the image quality [66]. They segmented pulmonary fissures using lung anatomy knowledge, which is time-consuming. There is a possibility that parts of fissures can be undetected due to the poorly segmented airways. To conclude, a more fine-grained fissure detection and lung lobe segmentation method will be ideal to pursue.

### 4.3. Report Generation.

Currently, most work applying knowledge graphs into report generation uses visual features for graph feature initializations. It is worth exploring different fusion methods to combine knowledge graphs with multimodal features. We can also encode and decode general knowledge for report generation tasks by exploring a general captioning framework guided by the auxiliary signals.

### 4.4. Image Retrieval.

In this review, two included articles regarding image retrieval used global and local indexing to infuse additional visual, textual, and knowledge graph features into image retrieval. One can further use appropriate clustering methods to take advantage of other fusion schemas. It is also worth exploring the visual filtering based on the local information from the semantic local indexing module to distill visual features for better performance.

## 5. Conclusions

This review discussed the current work on knowledge graph applications in medical imaging analysis and identified the limitations and future directions. We looked at the proven success of applying knowledge graphs into four medical imaging tasks: disease classification, disease localization and segmentation, report generation, and image retrieval. We identified the limitations due to limited annotated data for some supervised tasks and weak generalizability. We also identified potential future directions, for example, employing semisupervised framework, exploring different fusion methods, and exploring task-agnostic models that may improve the opportunities for better performance.

## Figures and Tables

**Table 1:** Search keywords to retrieve literature.

Database	Search keywords
IEEE Xplore, PubMed, Arxiv, Google Scholar, ACM Digital Library	Knowledge graph medical imaging OR knowledge graphs medical imaging OR knowledge graph medical image OR knowledge graphs medical image OR knowledge graph medical images OR knowledge graphs medical images OR graph medical imaging OR graphs medical imaging OR graph medical image OR graph medical images

**Table 2:** Overview of datasets used in the disease classification articles.

Ref	Year	Task	Dataset	Dataset info
[40]	2019	Binary classification	LIDC-IDRI [53]	1,018 chest CT scans with lung nodules present. CT scans were obtained from seven institutions
[45]	2020	Multilabel classification	IU X-ray [37]	3,955 radiology reports, 7470 chest X-ray images
[41]	2020	Binary classification	COVID-19CT report [54]	349 COVID-19 images and 379 non-COVID images and their corresponding Chinese reports
			Chest X-ray images (pneumonia) [55]	5,863 chest X-ray images with two classes: pneumonia and Normal
[50]	2020	Multilabel classification	CheXpert [39]	224,316 chest radiographs of 65,240 patients, with 14 common disease labels
			ChestX-Ray14 [38]	112,120 frontal-view X-ray images, with the text-mined 14 common disease labels
[46]	2021	Multilabel classification	IU X-ray [37]	3,955 radiology reports, 7470 chest X-ray images
			MIMIC-CXR [56]	377,110 chest X-ray images and the related 227,835 reports
[47]	2021	Multilabel classification	CheXpert [39]	224,316 chest radiographs of 65,240 patients, with 14 common disease labels
			NIH chest X-ray [38]	112,120 frontal-view X-ray images with the text-mined 14 common disease labels
[48]	2021	Multilabel classification	Chest ImaGenome [57]	242,072 images and the corresponding scene graphs
[42]	2021	Binary classification	Autism brain imaging data exchange (ABIDE) [58]	fMRI and the corresponding phenotypic data of 1,112 subjects
[49]	2021	Multilabel classification	CheXpert [39]	224,316 chest radiographs of 65,240 patients, with 14 common disease labels
[43]	2021	Binary classification	DDSM [59]	2,620 scanned film mammography studies.
[52]	2021	Binary classification and multilabel classification	COVID-19 [60], COVID-19 Radiography [61, 62]	150 CXR of COVID-19, 150 other pneumonia and another 150 instances for normal CXR images
[51]	2021	Binary classification	COVID-19 multimodal dataset	1,393 doctor-patient dialogues and 3706 images (347 X-ray +2,598 CT +761 ultrasound) about COVID-19 patients and 607 non-COVID-19 patient dialogues and 10,754 images (9658 X-ray + 494 CT +761 ultrasound)
[44]	2021	Multi-label classification	7PC [63]	1,011 lesion cases, and report comprehensive results

**Table 3:** Overview of datasets used in the disease localization and segmentation articles.

Ref	Year	Method	Dataset	Dataset info
[47]	2021	Visual spatial convolution, dual-weighting graph convolution	CheXpert [39] NIH chest X-ray [38]	224,316 chest radiographs of 65,240 patients and 14 common disease labels 112,120 X-ray images and the text-mined 14 common disease labels
[66]	2021	Fissure verification, surface fitting	LObe and Lung Analysis 2011 (LOLA11) [68]	A dataset of chest CT scans with varying abnormalities for which reference standards of lung and lobe segmentations have been established
[65, 67]	2021	U-Net [69]	NIH chest X-ray [38]	112,120 X-ray images and the text-mined 14 common disease labels

**Table 4:** Overview of datasets used in the report generation articles.

Ref	Year	Method	Dataset	Dataset info
[70]	2019	Graph transformer	CX-CHR dataset	Private dataset. 35,609 patients, 45,598 images and corresponding reports
			IU X-ray [37]	3,955 radiology reports, 7,470 chest X-ray images
[45]	2020	Two-level LSTM	IU X-ray [37]	3,955 radiology reports, 7,470 chest X-ray images
[71]	2020	Generative pretraining [75]	CX-CHR dataset	Private dataset. 35,609 patients, 45,598 images and corresponding reports
			COVID-19 CT report [54]	349 COVID-19 images, 379 non-COVID images and their corresponding Chinese reports
[43]	2021	Multihead attention, feed-forward network	IU X-ray [37]	3,955 radiology reports, 7,470 X-ray images
			MIMIC-CXR [56]	377,110 X-ray images, 227,835 reports

**Table 5:** Overview of datasets used in the image retrieval articles.

Ref	Year	Method	Dataset	Dataset info
[79, 80]	2006	Support vector machine (SVM)	Clef medical image database	50,000 medical images with the associated medical report in English, German, and French
